# DyLFG: A Dynamic Network Learning Framework Based on Geometry

**DOI:** 10.3390/e25121611

**Published:** 2023-11-30

**Authors:** Wei Wu, Xuemeng Zhai

**Affiliations:** 1 Changzhou College of Information Technology, Changzhou 213164, China; weiwucz@163.com; 2 School of Information and Communication Engineering, University of Electronic Science and Technology of China, Chengdu 611731, China

**Keywords:** temporal dynamics, hierarchical structure, geometry-based network representation

## Abstract

Dynamic network representation learning has recently attracted increasing attention because real-world networks evolve over time, that is nodes and edges join or leave the networks over time. Different from static networks, the representation learning of dynamic networks should not only consider how to capture the structural information of network snapshots, but also consider how to capture the temporal dynamic information of network structure evolution from the network snapshot sequence. From the existing work on dynamic network representation, there are two main problems: (1) A significant number of methods target dynamic networks, which only allow nodes to increase over time, not decrease, which reduces the applicability of such methods to real-world networks. (2) At present, most network-embedding methods, especially dynamic network representation learning approaches, use Euclidean embedding space. However, the network itself is geometrically non-Euclidean, which leads to geometric inconsistencies between the embedded space and the underlying space of the network, which can affect the performance of the model. In order to solve the above two problems, we propose a geometry-based dynamic network learning framework, namely DyLFG. Our proposed framework targets dynamic networks, which allow nodes and edges to join or exit the network over time. In order to extract the structural information of network snapshots, we designed a new hyperbolic geometry processing layer, which is different from the previous literature. In order to deal with the temporal dynamics of the network snapshot sequence, we propose a gated recurrent unit (GRU) module based on Ricci curvature, that is the RGRU. In the proposed framework, we used a temporal attention layer and the RGRU to evolve the neural network weight matrix to capture temporal dynamics in the network snapshot sequence. The experimental results showed that our model outperformed the baseline approaches on the baseline datasets.

## 1. Introduction

Networks are the ideal data structures to describe the interaction behavior of entities in complex systems that are ubiquitous in the real world. In recent years, network embedding [1,2,3,4] as a research hotspot has received more and more attention. The goal of network embedding is to map the nodes in the network to a low-dimensional space while keeping some information contained in the network. The resulting vector representations of nodes can be applied to a wide range of downstream tasks, such as community detection [5,6], node classification [7,8], link prediction [9,10], graph visualization [11], etc. With the rise of neural networks, people have put forward a variety of methods to use neural networks to deal with network information, so as to obtain informative network embedding. For example, Deepwalk [12] obtains local structural information about the network by truncating random walks and, then, processes this information using the skip-gram model to obtain the representations of nodes in the network. PRUNE [13] uses the Siamese neural network architecture [14] to process the global ranking information and local proximity information of the nodes to obtain the vector representations of the nodes in the network. Struc2vec [15] constructs the multi-layer network by defining the structural similarity between nodes and the weights of the edges between layers. Then, it uses the random walks on the multi-layer network to obtain the local and global structural information of the network, and then, it inputs this information into the neural network to acquire the representations of the nodes in the network. NANE [16] uses the fully connected layers and the autoencoder to process the structural information and attribute information sequentially to learn the network embedding, which can reflect the structural similarity and attribute similarity of nodes.

The models mentioned above focus on static networks; however, many real-world networks evolve dynamically over time, and the number of nodes and edges in these networks may change, as may the weights of the edges. For example, the emergence of new users in a social network will lead to the increase in the number of nodes, and the frequency of communication between the users will affect the weights of the edges. In general, a dynamic network can be represented as a collection of chronologically arranged network snapshots or as a network with timestamps on its edges. Here, we used the former representation. Compared with static-network-embedding methods, it is more challenging to find effective dynamic-network-embedding approaches. The reason is that these methods must not only capture the structural characteristics of the network, but more importantly, they must capture the dynamics of the network with respect to time, that is the change trend of the network snapshot structure. As a consequence, people start by capturing the structural and temporal characteristics of the network to solve the problem of dynamic network embedding. Dynnode2vec [17] is a dynamic-network-embedding method based on Node2vec [18], which is the static-network-embedding approach. It first uses Node2vec to obtain the embedding of the network snapshot at t = 0. When the network evolves over time, it only performs random walks in the region where the network evolves and, then, updates the embeddings of the nodes through the training of the skip-gram neural network model. DNE [19] proposes a decomposable objective function for learning the embedding of every node. When updating network embeddings, it only considers the newly added nodes and the most-affected old nodes. DynGEM [20] uses an autoencoder to learn the embedding of the dynamic network. Since the number of nodes in the network is not fixed, it uses a heuristic algorithm to dynamically adjust the size of the neural network. In addition, it uses the incremental learning method to generate the snapshot embedding of the current moment based on the snapshot embedding of the previous moment. Although the foregoing methods make use of the historical embeddings of the network in the learning process, their focus is to obtain the network embedding at the current moment, rather than the temporal dynamics or evolution trend of the network. Because the recurrent neural network (RNN) and its variants, such as long short-term memory (LSTM) and the GRU [21], can capture the temporal dynamics of a sequence of network snapshots, many neural network models use it as a component. For example, E-LSTM-D [22] adds an LSTM module to the autoencoder. The encoder–decoder architecture is responsible for extracting the network structure, and the LSTM module is used to capture the temporal dynamics during the evolution of the network structure. Different from the aforementioned methods, which use the RNN module to extract temporal dynamics, DySAT [23] and TSAM [24] use a temporal attention layer, that is an attention mechanism in the temporal dimension, which gives different snapshots different importance. The node selection strategy in GloDyNE [25] ensures the diversity of selected representative nodes, which can better maintain the global topology of the network. Then, the latest topology information of the selected nodes is encoded into the random walks, and then, the dynamic network embedding is obtained by combining with the skip-gram model.

The embedding spaces of the models mentioned above are all Euclidean spaces, and the networks themselves are non-Euclidean. Consequently, the geometric inconsistency between the network itself and the embedded space may restrict the performance improvement to some extent. Naturally, people try to use the more-general space, namely the manifolds, as the embedded spaces of the networks. Commonly used manifolds are the three kinds of constant curvature manifolds, namely Euclidean space, spherical space, and hyperbolic space, whose curvature is zero, positive, and negative, respectively. In recent years, some works [26,27,28] have been performed using hyperbolic geometry to model the networks. The hyperbolic space is a continuous version of a tree, so it has an advantage in modeling the hierarchical structure of the data, while the spherical space is suitable for modeling the cyclic structure in the data [29,30]. However, the existing work on network embedding involving constant curvature manifolds mainly focuses on static networks, and few works on dynamic network embedding have combined constant curvature spaces. For instance, SELFRGNN [31] proposed a Riemannian GNN with varying curvature, in which the Riemannian time encoding of arbitrary curvature is given, and a reweighted self-contrast method for self-supervised learning is combined to realize the representation learning of temporal graphs. The HVGNN [32] first proposed a temporal GNN based on the time-encoding method to model the dynamics of network sequences and, then, designed a hyperbolic graph variational autoencoder to model the uncertainty and generate the random node representation. It is worth noting that the simultaneous use of constant curvature manifolds and other non-Euclidean geometric tools to model dynamic networks from a geometric perspective is much rarer. In this paper, the hyperbolic geometry and Ricci curvature were used to model the structural information and temporal dynamics of the dynamic network snapshots. The main contributions of this paper are summarized as follows: (1) We propose a dynamic-network-learning framework, namely DyLFG, which uses geometric tools in non-Euclidean geometry to learn the embedding of dynamic networks. Our proposed framework targets dynamic networks, which allow nodes and edges to join or exit the network over time. (2) Our model uses the newly designed hyperbolic fully connected layers, which are different from previous work, to process the network snapshots, and it improves the ability to extract network structural information due to the matching of the underlying geometry. (3) We modified the GRU module with the Ricci curvature to improve the ability of the GRU to extract the temporal dynamics of the network snapshot sequence. (4) A number of experiments were conducted on the benchmark datasets to compare the performance of DyLFG with the baseline approaches.

## 2. Background and Related Work

In this section, we review the basic concepts related to the hyperbolic geometry and Ricci curvature, as well as some related work in the current literature on dynamic networks. Firstly, we briefly review the characteristics and common models of the hyperbolic geometry and, then, introduce some useful geometric operations in the hyperbolic space. Secondly, we introduce the concept of the Ricci curvature related to the Wasserstein distance. Finally, we discuss some of the work on dynamic networks in the existing literature.

### 2.1. Basic Conceptions of Hyperbolic Space

From the Introduction of the first section, we already know that the hyperbolic space is a negative curvature manifold. Due to the property of the negative curvature, a hyperbolic space is intuitively larger than a Euclidean space, which prevents it from being embedded as a subset into a Euclidean space. Consequently, many equivalent models of the hyperbolic space have been developed. Each model focuses on some aspects of the hyperbolic geometry, but no model can express all the properties of the hyperbolic geometry. One of these models is the hyperboloid model, which is used later in this paper to represent the hyperbolic space. More details on these models can be found in [33,34].

**Definition 1**  ([26,28])**.** *The hyperboloid model $M=H^{n}$ of the hyperbolic space is defined as*

(1) $$H^{n}=\left\{u\in R^{n+1}:<u,u{>}_{M}=-1,u_{0}>0\right\}.$$

*where $<\cdot ,\cdot {>}_{M}$ is the Minkowski inner product of the form:*

(2) $$<u,v{>}_{M}=-u_{0}v_{0}+\sum_{i=1}^{n}u_{i}v_{i}$$

*for $u,v\in R^{n+1}$, and the distance metric in $H^{n}$ is*

(3) $$d_{M}\left(u,v\right)=arccosh\left(-<u,v{>}_{M}\right),$$

*for $u,v\in H^{n}$.*

The above definition gives the concept of the hyperbolic space. However, it should be noted that a hyperbolic space is a manifold, not a vector space, which causes two elements in a hyperbolic space to be unable to perform operations such as addition like their counterparts in a vector space. To perform operations such as addition, it is necessary to use the following concept of the tangent space to convert elements in the hyperbolic space into vectors in the tangent space, and the tangent space is a vector space, which can perform operations such as addition; see Section 2.2 for details.

**Definition 2**  ([26,28])**.** *The tangent space of $H^{n}$ at point p is a Euclidean space, which is defined as*

(4) $$T_{p}H^{n}=\left\{u\in R^{n+1}:<u,p{>}_{M}=0\right\},$$

*and the norm of $u\in T_{p}H^{n}$ is defined as*

(5) $${∥u∥}_{M}=\sqrt{<u,u{>}_{M}}.$$

### 2.2. Geometric Operations of Hyperbolic Space

**Figure 1:**
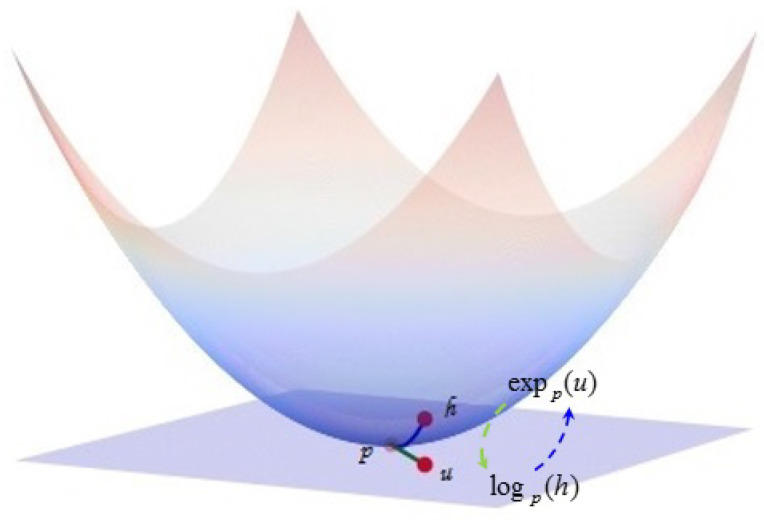
Illustration of exponential and logarithmic maps.

Since the hyperbolic space is a manifold rather than a vector space, for optimization involving the hyperbolic space, it is necessary to switch between the hyperbolic space, tangent space, and Euclidean space embedded in the hyperbolic space. This requires the exponential map, logarithmic map, and projection operation described below. As shown in Figure 1, the function of the exponential map is to map points in the corresponding tangent space to the hyperbolic space, and the function of the logarithmic map is to map points in the hyperbolic space to the corresponding tangent space, that is the switch between the corresponding points in the hyperbolic space and the tangent space can be realized through these two operations. To map points in the Euclidean space outside the hyperbolic space into the hyperbolic space, it is necessary to use the projection operation. In particular, it is important to note that such points may not be in the tangent space of a hyperbolic space, so the logarithmic map cannot replace the projection operation. In order to multiply the weight matrix and the point p in the hyperbolic space, in Equation (8), the point p is first mapped to the corresponding tangent space by the logarithmic map. Since the tangent space is a Euclidean space, it is possible to multiply the weight matrices W and $log_{o}\left(p\right)$ and, then, map the resulting new vector back into the hyperbolic space through an exponential map. This is the geometric essence behind the concept of the manifold linear transformations given below. Next, we give the definitions of these geometric operations [28].

**Logarithmic and exponential map.**  For $p,h\in M=H^{n}$ and $u\in T_{p}M$, the exponential map of the manifold M is defined as

(6) $$exp_{p}\left(u\right)={cosh(∥u∥}_{M}{)p+sinh(∥u∥}_{M})\frac{u}{{∥u∥}_{M}},$$

and the logarithmic map is given by

(7) $$log_{p}\left(h\right)=d_{M}\left(p,h\right)\frac{h+<p,h{>}_{M}p}{∥h+<p,h{>}_{M}{p∥}_{M}}.$$

**Remark:**  Figure 1 shows the exponential and logarithmic maps of a hyperbolic space. It is easier to understand the definitions of these two maps in conjunction with Figure 1.

**Manifold linear transform:**  As mentioned above, operating on points on the manifold requires the aid of tangent spaces. The construction of a neural network usually involves multiplying a matrix by a vector and adding a bias vector. The definitions of these operations on manifolds are different from those of Euclidean spaces, as follows:

(8) $$W⊗p=exp_{o}\left(W\cdot log_{o}\left(p\right)\right),$$

where *p* is a point in the manifold M and *W* is the weight matrix, and the tangent space involved in logarithmic and exponential map is the tangent space at the origin.

**Projection:**  The projection operation is performed to project the vector onto the hyperboloid and the corresponding tangent space, which is useful for the optimization process. Let $p=\left(p_{0},p_{1:n}\right)\in R^{n+1}$, then it can be projected on the hyperboloid space $H^{n}$ as follows:

(9) $$Proj_{H^{n}}\left(p\right)=\left(\sqrt{1+∥p_{1:n}{∥}_{2}^{2}},p_{1:n}\right).$$

where ${∥\cdot ∥}_{2}$ is the Euclidean norm.

### 2.3. Ricci Curvature

In a probability density space defined on the metric space of a network, a natural Riemannian metric can be assigned to the probability density space through the Wasserstein distance [35]. In [36], the Wasserstein distance was used to define the Ricci curvature of an edge in the network, which can reflect the similarity between two nodes connected by the edge.

**Definition 3**  ([36])**.** *Suppose G is an undirected and unweighted network. Then, the probability measure of node u is defined as*

(10) $$m_{u}\left(v\right)=\left\{\begin{array}{cc} \frac{1}{d_{u}}, & if\;v\in \Gamma (u)\\ 0, & otherwise, \end{array}\right.$$

*where $d_{u}$ is the degree of node u and Γ(u) is the set of neighbors of u. Further, Let $m_{u},m_{v}$ be two probability measures conforming to the Equation* (10). *Then, the Wasserstein distance* [37] *between two measures is defined as*

(11) $$W\left(m_{u},m_{v}\right)=\underset{\Pi }{\inf}\sum_{u^{\prime }\in \Gamma \left(u\right)}\sum_{v^{\prime }\in \Gamma \left(v\right)}d\left(u^{\prime },v^{\prime }\right)\Pi \left(u^{\prime },v^{\prime }\right),$$

*where d(u,v) is the shortest path length between the nodes u,v, and the matrix* Π *should satisfy the following conditions:*

(12) $$\sum_{u^{\prime }\in \Gamma \left(u\right)}\Pi \left(u^{\prime },v^{\prime }\right)=m_{v}\left(v^{\prime }\right),\sum_{v^{\prime }\in \Gamma \left(v\right)}\Pi \left(u^{\prime },v^{\prime }\right)=m_{u}\left(u^{\prime }\right).$$

Consequently, we can utilize the Wasserstein distance to define the Ricci curvature of the edges in the network.

**Definition 4**  ([36,38])**.** *Let G be an undirected and unweighted network, then the Ricci curvature of edge (u,v) is defined as*

(13) $$\kappa \left(u,v\right)=1-W\left(m_{u},m_{v}\right),$$

*where u,v are the nodes of network G.*

**Figure 2:**
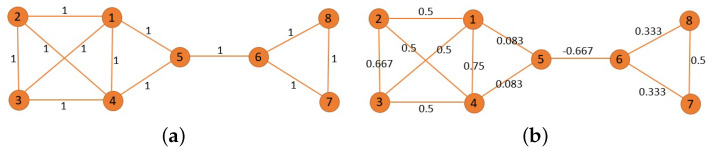
The Ricci curvature with examples. (**a**) A network with all edge weights of 1; (**b**) a demonstration of the Ricci curvature of the network in (**a**).

In order to establish an intuitive understanding of the Ricci curvature, we take the toy network in Figure 2 as an example. Figure 2a shows a network with all edge weights of one, and Figure 2b is obtained by replacing the edge weights of the aforementioned network with the corresponding Ricci curvature from the original value. It is easy to find that the network corresponding to Figure 2a has an obvious community structure, that is it is composed of two communities. One community in this network consists of nodes 1, 2, 3, 4, and 5, while the other community consists of nodes 6, 7, and 8. The edge weights in Figure 2a clearly do not reflect the meso-level hierarchy of the network, that is the community. But, that is where the Ricci curvature comes in. For example, the Ricci curvature of the edge (5, 6) in Figure 2b is negative, and the Ricci curvature of the edges between communities in the network often has such a negative property. In addition, for an edge with a positive Ricci curvature, the greater the value, the closer the relationship between the two nodes connected by the edge is.

**Figure 3:**
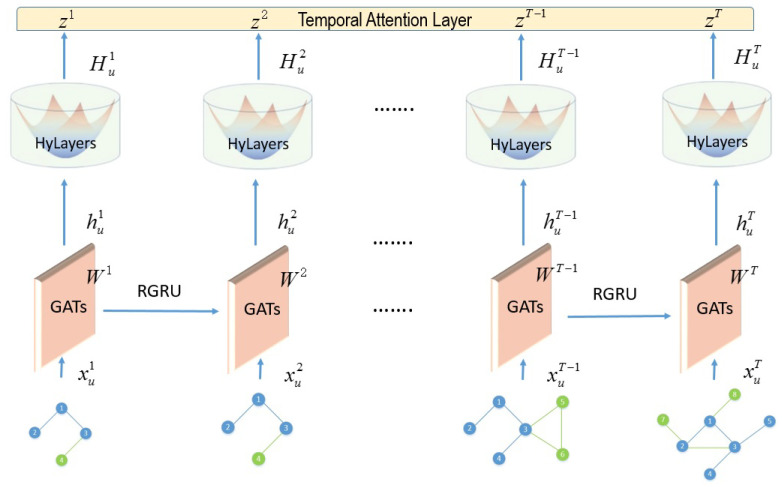
Architecture of the model DyLFG.

Here, we highlight the connection between the background knowledge and the proposed DyLFG model. Referring to the DyLFG model architecture diagram in Figure 3, the concepts and geometric operations related to the hyperbolic space in Section 2.1 and Section 2.2 are closely related to the HyLayers module in the second layer of the model, which will use a newly designed hyperbolic operation method different from the previous one. For the details, see Equations (19) and (20). The concept of the Ricci curvature in Section 2.3 is closely related to the RGRU module in the first layer of the model, specifically referring to Equations (17) and (18). Because the RGRU module, which is Equation (18), uses the adjacency matrix information and Ricci curvature matrix information of the network snapshot at the same time, the advantages of the Ricci curvature reflecting the community structure and node similarity measurement can be better utilized in the aggregation of the information.

### 2.4. Related Work

The methods of static networks mainly realize embeddings by using network topology information and attribute information, while the approaches of dynamic networks should pay extra attention to the temporal dynamics inherently contained in the network snapshots. People try to use various methods to model the temporal dynamics while capturing the network structure characteristics, so that the model can capture the trend of network evolution. Dyrep [39] uses the temporal point process model defined by the intensity function to simulate the events occurring in the network and obtains the network embedding through the training of the neural network. HTNE [40] integrated the embeddings of the nodes into the conditional intensity function of the Hawkes process and, then, obtained the embedding of dynamic network by optimizing the likelihood function built on the basis of the intensity function. EvolveGCN [41] uses a two-layer GCN model to simulate the network structure and, then, evolves the parameters of each GCN layer through the RNN to capture the temporal dynamics. In this way, it integrates the learning of the spatial dimension and temporal dimension together. DySAT [23] uses the GAT [42] model to extract the structural features of the network snapshots and, then, weights the outputs of the GAT at different time steps through the temporal attention mechanism to embrace the dynamics in the node embeddings.

## 3. Problem Definition

A dynamic network *G* can be represented as a sequence of network snapshots, i.e., $G=\{G_{1},G_{2},\cdots ,G_{T}\}$, where *T* is the number of time steps. Each snapshot $G_{t}=\left(V_{t},E_{t}\right)$ is a weighted and undirected network with vertex set $V_{t}$ and edge set $E_{t}$, which is represented by the adjacency matrix $A^{t}\in R^{|V_{t}\left|\times \right|V_{t}|}$, where $|V_{t}|$ represents the number of nodes in the network snapshot $G_{t}$. It should be noted that, unlike some previous approaches that require nodes or edges of the dynamic network to increase over time, but not decrease over time, the nodes and edges of the dynamic network analyzed by the framework in this paper are not restricted to change over time, that is nodes can be added or deleted over time, and edges can also be added or deleted over time. For the convenience of processing, the adjacency matrix of all snapshots is expanded to a new adjacency matrix ${\tilde{A}}^{t}$ of the same size *N* by zero-padding as follows, where $V=\overset{T}{\underset{t=1}{⋃}}V_{t}$ is a shared node set, N=|V| is the cardinality of the shared node set V, and ${\tilde{A}}^{t}\in R^{N\times N}$ is the new adjacency matrix of network snapshot $G_{t}$.

(14) $${\tilde{A}}^{t}=\left\{\begin{array}{cc} A_{ij}^{t}, & i\in V_{t}\;and\;j\in V_{t}\\ 0, & i\in V\setminus V_{t}\;or\;j\in V\setminus V_{t} \end{array}\right.$$

As can be seen from the definition of Equation (14), ${\tilde{A}}^{t}$ is mainly to transform the adjacency matrix of all network snapshots to the same size, and it is essentially no different from the original adjacency matrix $A^{t}$ of network snapshots. The goal of dynamic network representation learning is to obtain the embedding $z_{u}^{t}$ of each node *u* at time step *t*, so that $z_{u}^{t}$ can not only preserve the structural information of snapshot corresponding to time *t*, but also capture the temporal evolution trend implied by structural changes of all snapshots before time *t*.

## 4. Methods

Based on the Ricci curvature and hyperbolic geometry, we propose a dynamic-network-embedding method called DyLFG from a geometric point of view. As shown in Figure 3, the model DyLFG is mainly composed of 3 modules. The first module is the GAT stack layers, connected in series with the RGRU; the second is the newly designed hyperbolic module called HyLayers, which is used to hierarchically sort out the hidden layer representation of the network data; the third is time attention module, which is used to capture the time dynamics. One of the main innovations of this paper is to use the Ricci-curvature-based GRU module, namely the RGRU, to connect the GAT modules in series, so that the GAT modules can not only extract the structural information of their respective network snapshots in isolation, but also capture the evolution trend of the network snapshot structure over time. In addition, another innovation is that, in the hyperbolic module, the DyLFG uses multiple hyperbolic layers different from those in the previous literature to arrange the data hierarchically. At the end of this section, we give a DyLFG model based on the above functional modules in structural information or temporal dynamics extraction.

### 4.1. GAT Stacked Module and RGRU

In this section, we specify how the GAT and RGRU modules in Figure 3 are constructed. The input to the first GAT layer is the set of node representations in the network snapshot $G_{t}$, denoted $\left\{x_{u}^{t}\in R^{D},u\in V\right\}$ with dimensionality *D*, which can be initialized with the one-hot vectors. Note that the input was unified using zero-padding to the same dimension *D* as the cardinality of the shared node set *V*. The output of this layer is $\left\{h_{u}^{t,(1)}\in R^{F_{1}},u\in V\right\}$ with dimensionality $F_{1}$, which is obtained by using the attention mechanism to process the structural information in the one-hop neighborhood of the nodes in the network snapshot $G_{t}$. In order to expand the scope of exploring the structure of the network snapshot, a GAT layer with the same structure is stacked on top of the first GAT layer. The input of the second GAT layer is the output of the first GAT layer, namely $\left\{h_{u}^{t,(1)}\in R^{F_{1}},u\in V\right\}$ with dimensionality $F_{1}$, and the output of the second GAT layer is $\left\{h_{u}^{t}\in R^{F_{1}},u\in V\right\}$ with dimensionality $F_{1}$. The two GAT layers are defined as follows:

(15) $$\left\{\begin{array}{cc} \gamma_{uv}^{k,t,(1)} & =LeakyReLU({\underline{A}}_{uv}^{t}\cdot {\left(a^{k,(1)}\right)}^{T}\left[W^{k,(1)}h_{u}^{t,(0)}∥W^{k,(1)}h_{v}^{t,(0)}\right])\\ \beta_{uv}^{k,t,(1)} & =\frac{exp(\gamma_{uv}^{k,t,(1)})}{\sum_{w\in \Gamma (v)}exp\left(\gamma_{wv}^{k,t,(1)}\right)}\\ h_{u}^{k,t,(1)} & =LeakyReLU(\sum_{u\in \Gamma (v)}\beta_{uv}^{k,t,(1)}W^{k,(1)}h_{u}^{t,(0)})\\ h_{u}^{t,(1)} & {=∥}_{k=1}^{K}h_{u}^{k,t,(1)} \end{array}\right.$$

(16) $$\left\{\begin{array}{cc} \gamma_{uv}^{k,t,(2)} & =LeakyReLU({\underline{A}}_{uv}^{t}\cdot {\left(a^{k,(2)}\right)}^{T}\left[W^{k,t,(2)}h_{u}^{t,(1)}∥W^{k,t,(2)}h_{v}^{t,(1)}\right])\\ \beta_{uv}^{k,t,(2)} & =\frac{exp(\gamma_{uv}^{k,t,(2)})}{\sum_{w\in \Gamma (v)}exp\left(\gamma_{wv}^{k,t,(2)}\right)}\\ h_{u}^{k,t,(2)} & =Tanh(\sum_{u\in \Gamma (v)}\beta_{uv}^{k,t,(2)}W^{k,(2)}h_{u}^{t,(1)})\\ h_{u}^{t} & {=∥}_{k=1}^{K}h_{u}^{k,t,(2)} \end{array}\right.$$

where $h_{u}$ is the hidden representation of node *u* and $h_{v}$ is the hidden representation of node *v*, where nodes *u* and *v* are associated with edges (u,v). Here, *K* represents the number of attention heads of the attention mechanism in the GAT layer, ∥ represents the concatenation operation, superscript *t* represents the corresponding tth network snapshot, superscript *k* represents the coefficient or vector corresponding to the *k*th attention head, $\gamma_{uv}^{k,t,(i)}$ and $\beta_{uv}^{k,t,(i)}$, respectively, represent the unnormalized and normalized attention coefficients corresponding to the kth attention head in the ith GAT layer, $h_{u}^{t,(0)}=x_{u}^{t}\in R^{D}$ with dimensionality *D*, $W^{k,(1)}$ is the shared weight matrix corresponding to the kth attention head of the first GAT layer applied to each network snapshot, $W^{k,t,(2)}$ is the non-shared weight matrix corresponding to the kth attention head in the second GAT layer applied to the network snapshot $G_{t}$, ${\underline{A}}^{t}=D^{-\frac{1}{2}}\left(A^{t}+I\right)D^{-\frac{1}{2}}$, *I* is the identity matrix, $D_{ii}=\sum_{i}{\left(A^{t}+I\right)}_{ij}$, and ${\underline{A}}_{uv}^{t}$ is the element value corresponding to nodes *u* and *v* in the adjacent matrix of network snapshot $G_{t}$.

According to Equation (15), the embedding dimension of each node in different network snapshots is the same. However, considering that the number of nodes in each network snapshot is generally different, the embedding matrix or tensor formed by stacking all the nodes in each network snapshot together is generally different in the number of rows and the same in the number of columns. In order to obtain an embedding matrix of uniform size, we selected the shared node set of all network snapshots, and the cardinality of the shared node set is the maximum node number *N*. Then, we compared the node set of each network snapshot with the shared node set, manually added the non-existent node (the node does not have any edge in the network), and gave the node all-0 embedding. This operation is to perform zero-padding on the embedding matrix of the network snapshot, so that the size of the embedding matrix corresponding to all network snapshots is unified. The embedding matrix obtained by 0-filling expansion is denoted as $h^{t,(1)}$.

The previous GAT stack module can capture the structural information of the corresponding network snapshot, but cannot capture the network change trend information contained in the network snapshot sequence. To capture this time-related trend information, the DyLFG model uses a newly designed RGRU module, which is a different RNN variant of the previous GRU module based on the Ricci curvature. The RGRU module is mainly used to evolve the weight matrix of the second-layer GAT of the GAT stack module, thus connecting the GAT modules corresponding to each time step in series and mining the time dynamics in it. The derivation formula of the schematic property of the RGRU module is given by Equation (17).

(17) $$\left\{\begin{array}{cc} \; & W^{t-1}{=∥}_{k=1}^{K}W^{k,t-1,(2)}\\ \; & W^{t}=RGRU\left(h^{t,(1)},W^{t-1}\right)\\ \; & Set\left(W^{k,t,(2)}\right)=split\left(W^{t}\right) \end{array}\right.$$

The first formula in Equation (17) is to concatenate the weight matrix $W^{k,t-1,(2)}$ corresponding to all *K* attention heads of the t−1th time step in Equation (16) to obtain the total weight matrix $W^{t-1}$ for this time step. Then, using the input $h^{t,(1)}$ of the second-layer GAT in the *t* time step and the weight matrix $W^{t-1}$ of the t−1th time step, the weight matrix $W^{t}$ of step *t* is obtained. Consequently, the weight matrices of the second-layer GAT corresponding to the adjacent time steps are linked together. Combined with the obtained preliminary network structure coding information, the time evolution information of the network snapshot sequence is explored in this way at the bottom of the DyLFG model. Further temporal trend mining will take place at the top of the DyLFG model. Finally, in the third formula of Equation (17), $W^{t}$ is split into a set of small weight matrices $W^{k,t,(2)}$ corresponding to *K* attention heads, respectively.

The second formula in Equation (17), which is the specific calculation formula of the RGRU, is as follows:

(18) $$\left\{\begin{array}{c} Z_{t}=Sigmoid\left[D_{t}^{-\frac{1}{2}}\left(\lambda_{1}{\tilde{\underline{A}}}^{t}+\lambda_{2}{\tilde{\underline{A}}}_{c}^{t}\right)D_{t}^{-\frac{1}{2}}W_{z}h^{t,(1)}+D_{t}^{-\frac{1}{2}}\left(\lambda_{1}{\tilde{\underline{A}}}^{t}+\lambda_{2}{\tilde{\underline{A}}}_{c}^{t}\right)D_{t}^{-\frac{1}{2}}U_{z}W^{t-1}+B_{z}\right]\\ R_{t}=Sigmoid\left[D_{t}^{-\frac{1}{2}}\left(\lambda_{1}{\tilde{\underline{A}}}^{t}+\lambda_{2}{\tilde{\underline{A}}}_{c}^{t}\right)D_{t}^{-\frac{1}{2}}W_{r}h^{t,(1)}+D_{t}^{-\frac{1}{2}}\left(\lambda_{1}{\tilde{\underline{A}}}^{t}+\lambda_{2}{\tilde{\underline{A}}}_{c}^{t}\right)D_{t}^{-\frac{1}{2}}U_{r}W^{t-1}+B_{r}\right]\\ {\tilde{h}}_{t}=Tanh\left[D_{t}^{-\frac{1}{2}}\left(\lambda_{1}{\tilde{\underline{A}}}^{t}+\lambda_{2}{\tilde{\underline{A}}}_{c}^{t}\right)D_{t}^{-\frac{1}{2}}W_{m}h^{t,(1)}+D_{t}^{-\frac{1}{2}}\left(\lambda_{1}{\tilde{\underline{A}}}^{t}+\lambda_{2}{\tilde{\underline{A}}}_{c}^{t}\right)D_{t}^{-\frac{1}{2}}U_{m}R_{t}⊙W^{t-1}+B_{m}\right]\\ W^{t}=\left(1-Z_{t}\right)⊙W^{t-1}+Z_{t}⊙{\tilde{h}}_{t} \end{array}\right.$$

where $\lambda_{1}$ and $\lambda_{2}$ are hyperparameters, $h^{t,(1)}$ is the embedding matrix of the time step *t* obtained by 0-filling the output of the first-layer GAT mentioned earlier, $W^{t-1}$ is the weight matrix corresponding to the second GAT time step t−1, $\left\{W_{j},U_{j},B_{j}\right\},j=\left\{z,r,m\right\}$ are learnable parameters, ${\tilde{A}}_{c}^{t}$ is the curvature matrix of uniform size obtained by treating the Ricci curvature matrix of the network snapshot with *t* time steps by the similar Equation (14), ⊙ represents the Hadamard product of two matrices, and Tanh(·) and Sigmoid(·) are the tanh and sigmoid activation function. It should be noted that, in order to avoid the adverse effects of negative values in the original Ricci curvature, the curvature in ${\tilde{A}}_{c}^{t}$ is the value of the original curvature processed by the sigmoid function. Here, ${\tilde{\underline{A}}}^{t}=D^{-\frac{1}{2}}\left({\tilde{A}}^{t}+I\right)D^{-\frac{1}{2}}$, and *I* is the identity matrix, $D_{ii}=\sum_{i}{\left({\tilde{A}}^{t}+I\right)}_{ij}$. Similarly, ${\tilde{\underline{A}}}_{c}^{t}=D^{-\frac{1}{2}}\left({\tilde{A}}_{c}^{t}+I\right)D^{-\frac{1}{2}}$, *I* is the identity matrix, $D_{ii}=\sum_{i}{\left({\tilde{A}}_{c}^{t}+I\right)}_{ij}$. $D_{t}$ is a diagonal matrix whose diagonal elements are the sum of each row of $\lambda_{1}{\tilde{\underline{A}}}^{t}+\lambda_{2}{\tilde{\underline{A}}}_{c}^{t}.$

### 4.2. Hyperbolic Geometric Transition Layer

Considering the natural hierarchical characteristics of network data and the advantages of the hyperbolic space for hierarchical data embedding, we newly designed a different hyperbolic module to re-encode the hidden layer representation output by the previous GAT module. The hyperbolic module consists of two hyperbolic fully connected layers with decreasing output dimensions in the hope that a more-compact representation can better capture hidden patterns in the data. The specific formula of the hyperbolic module is as follows:

(19) $$\left\{\begin{array}{cc} {\tilde{W}}_{h}^{1} & =log_{0}\left(Proj_{H^{n}}\left(W_{h}^{1}\right)\right)\\ {\tilde{H}}^{t,(1)} & =log_{0}\left(Proj_{H^{n}}\left(h^{t}\right)\right)\\ H^{t,(1)} & =LeakyReLU(exp_{0}\left({\tilde{H}}^{t,(0)}\cdot {\tilde{W}}_{h}^{1}\right)) \end{array}\right.$$

where ${\tilde{W}}_{h}^{1}$,${\tilde{H}}^{t,(0)}$ are obtained, respectively, by projecting the weight matrix $W_{h}^{1}\in R^{F_{1}\times F_{2}}$ into the Euclidean space and the output $h^{t}\in R^{F_{2}}$ of the GAT module into the hyperbolic space and, then, pulling back to the tangent space. Here, $F_{1}$ and $F_{2}$ are the corresponding dimensions. The third formula in Equation (19) is to multiply ${\tilde{W}}_{h}^{1}$ and ${\tilde{H}}^{t,(0)}$ in the tangent space and map back to the hyperbolic space before using the LeakyReLU activation function for the nonlinear transformation.

(20) $$\left\{\begin{array}{cc} {\tilde{W}}_{h}^{2} & =log_{0}\left(Proj_{H^{n}}\left(W_{h}^{2}\right)\right)\\ {\tilde{H}}^{t,(1)} & =log_{0}\left(Proj_{H^{n}}\left(H^{t,(1)}\right)\right)\\ H^{t} & =Tanh(exp_{0}\left({\tilde{H}}^{t,(1)}\cdot {\tilde{W}}_{h}^{2}\right)) \end{array}\right.$$

where ${\tilde{W}}_{h}^{2}$,${\tilde{H}}^{t,(1)}$ are obtained, respectively, by projecting the weight matrix $W_{h}^{2}\in R^{F_{2}\times F}$ of the Euclidean space and the output $H^{t,(1)}\in R^{F}$ of the first hyperbolic fully connected layer into the hyperbolic space and, then, pulling back to the tangent space by logarithmic mapping. Here, $F_{2}$ and *F* are the corresponding dimensions. The third formula in Equation (20) is used to multiply ${\tilde{W}}_{h}^{2}$ and ${\tilde{H}}^{t,(1)}$ in the tangent space and, then, map back to the hyperbolic space with an exponential mapping followed by a nonlinear transformation using the activation function tanh.

### 4.3. Temporal Attention Layer

Similar to DySAT [23], DyLFG uses a temporal attention network, which uses a multi-head attention mechanism. The role of the temporal attention network is to extract temporal dynamics from the input snapshot sequence, which gives different importance to the elements in the snapshot sequence. Specifically, the input representation of node *u* at each time step is $H_{u}=\left\{H_{u}^{1},H_{u}^{2},\cdots ,H_{u}^{T}\right\}$, where *T* is the number of time steps, $H_{u}^{t}\in R^{F}$. In the output of the temporal attention network, the representation of node *u* at all time steps is $y_{u}=\left\{y_{u}^{1},y_{u}^{2},\cdots ,y_{u}^{T}\right\}$, where $y_{u}^{t}\in R^{F}$, and *F* is the dimension of the node in the output. We arranged the representations of node *u* in the input and output in time and stacked them together, which are denoted as $H_{u}\in R^{T\times F}$ and $Y_{u}\in R^{T\times F}$. Here, *T* is the number of time steps in the dynamic network and *F* is the corresponding dimension.

Here, the temporal attention network is constructed by the attention mechanism expressed in [43]. First, linear transformations are used to process the input vector to obtain the queries, keys, and values, i.e.,

(21) $$Q=H_{u}W_{q},K=H_{u}W_{k},V=H_{u}W_{u},$$

where $W_{q},W_{k},W_{u}\in R^{F\times F}$ are the learnable weights. Then, the temporal attention network is constructed as follows:

(22) $$\left\{\begin{array}{cc} y_{u}^{k} & =\alpha_{u}V\\ \alpha_{u}^{ij} & =\frac{exp(r_{u}^{ij})}{\sum_{k=1}^{T}exp\left(r_{u}^{ik}\right)}\\ r_{u}^{ij} & =\frac{{\left(QK^{T}\right)}_{ij}}{\sqrt{F}}+M_{ij} \end{array}\right.$$

where $\alpha_{u}$ is the attention weight matrix, and $M_{ij}=0$ when i≤j, otherwise −∞.

After generating the node embedding corresponding to each attention head, we only need to concatenate these embedding vectors together to obtain the node embedding of multiple attention heads. The principle of structural multi-head attention is the same as that of temporal multi-head attention. Next, we use a common form to illustrate their attention mechanisms. The embedded vector corresponding to each attention head of node *u* is denoted as $\left\{y_{u}^{1},y_{u}^{2},\cdots ,y_{u}^{K}\right\}$, where *K* is the total number of attention heads. Consequently, the output vector $y_{u}$ of the multi-head attention is calculated as follows:

(23) $$y_{u}=Concat\left(y_{u}^{1},y_{u}^{2},\cdots ,y_{u}^{K}\right)$$

where Concat(·) represents the concatenation of the vectors.

### 4.4. DyLFG Architecture

The DyLFG framework and the construction of each module are described in detail above. The lower layer of the DyLFG model consists of the GAT module, and the GAT module consists of 2 layers of the GAT. One of the roles of these GAT layers is to extract low-order and high-order structural information from the current network snapshot. Considering the geometric fit between the hyperbolic space and the hierarchy of the network data, a new hyperbolic fully connected module is designed in the DyLFG model to re-encode the hidden layer representation of the GAT module output. In order to capture the temporal dynamics contained in the network snapshot sequence, the DyLFG model, on the one hand, uses the newly designed RGRU module to evolve the weight matrix in the second GAT layer at the lower level of the model to achieve this purpose, and on the other hand, it uses the temporal attention layer at the top level of the model to process the coding information to further extract the evolution trend of the network snapshot sequence. Next, each component of the DyLFG model is described in detail.

As shown in Figure 3, network snapshots at each time step are input into the DyLFG model. Specifically, the inputs are the adjacency matrix $A^{t}$ and the feature vectors of the nodes in the snapshots. Then, after processing by the first GAT layer of the GAT module, the structural information of each time step network snapshot is extracted, and the code $\left\{h_{u}^{1,(1)},h_{u}^{2,(1)},\cdots ,h_{u}^{T,(1)}\right\},h_{u}^{t,(1)}\in R^{F_{1}}$ of the first hidden layer is output. This code is then entered into the second GAT layer of the GAT module, whose output is $\left\{h_{u}^{1},h_{u}^{2},\cdots ,h_{u}^{T}\right\},h_{u}^{t}\in R^{F_{1}}$. This GAT layer differs from the previous one in that it uses a newly designed RGRU module to concatenate and evolve the weight matrix of the GAT over time to further capture higher-order structural information and temporal dynamics. In view of the hierarchical structure of the network data and the advantages of the hyperbolic space for hierarchical data modeling, the hyperbolic module was designed to re-encode the output of the GAT module, which is different from the previous ones in the literature. The hyperbolic module receives the output of the GAT module as the input, and the output of its first hyperbolic fully connected layer is $\left\{H_{u}^{1,(1)},H_{u}^{2,(1)},\cdots ,H_{u}^{T,(1)}\right\},H_{u}^{t,(1)}\in R^{F_{2}}$. It should be noted that $F_{2}$ is less than $F_{1}$, that is the hyperbolic layer compresses the encoding dimension of the hidden layer. In order to better capture the patterns contained in the structural information by further compressing the dimensions represented by the hidden layer, a similar hyperbolic fully connected layer is stacked on top of the previous hyperbolic fully connected layer, but with a different activation function and a smaller dimension. The output of this layer is $\left\{H_{u}^{1},H_{u}^{2},\cdots ,H_{u}^{T}\right\},H_{u}^{t}\in R^{F}$. In order to further capture the evolution trend contained in the network snapshot sequence, a temporal attention layer is deployed on top of the DyLFG model. The output of this layer, namely the final output of the DyLFG model, is $\left\{y_{u}^{1},y_{u}^{2},\cdots ,y_{u}^{T}\right\},y_{u}^{t}\in R^{F}$. Here, *T* is the number of time steps in the dynamic network, while $F_{1}$, $F_{2}$, and *F* are the corresponding dimensions.

Next, we constructed the loss function of the DyLFG model based on the output of temporal attention layers, namely $\left\{y_{u}^{1},y_{u}^{2},\cdots ,y_{u}^{T}\right\}$ with the number of time steps *T*. One of the basic ideas of network embedding is that similar nodes are close to each other in the embedding space, while dissimilar nodes are far away from each other. We perform random walks on the network snapshots and, then, use the sliding window to find the generalized neighbors of the target nodes. As positive samples, these neighbors are required to be close to each other in the embedded space. Then, the negative samples are sampled according to the common negative sampling distribution, and they are required to be far away from each other in the embedded space.

Due to the use of the newly designed RGRU module to evolve the GAT’s weight matrix and time attention layer processing, $\left\{y_{u}^{1},y_{u}^{2},\cdots ,y_{u}^{T}\right\}$ contains the temporal dynamics in the network snapshot sequence, where *T* is the number of time steps in the dynamic network. We require this representation to preserve the local structural information of the snapshot at each time step. Consequently, the loss function here is defined as

(24) $$L_{1}=\sum_{t=1}^{T}\delta^{t}\sum_{u\in V}\left(\sum_{v\in N^{t}\left(u\right)}-log\left(\sigma \left(<y_{v}^{t},y_{u}^{t}>\right)\right)-\gamma \sum_{v^{\prime }\in P_{neg}^{t}\left(u\right)}log\left(1-\sigma \left(<y_{v^{\prime }}^{t},y_{u}^{t}>\right)\right)\right).$$

where $N^{t}\left(u\right)$ is the generalized neighborhood of the node *u* in the truncated random walk on snapshot $G_{t}$, $P_{neg}^{t}\propto d_{u}^{\mu }$ is the negative sampling distribution of snapshot $G_{t}$, μ is a constant, $d_{u}$ is the degree of node u, γ is the weighting coefficient of the loss corresponding to negative samples, *T* is the number of time steps in the dynamic network, and σ is the sigmoid activation function. δ is the attenuation constant, which is set for the purpose of minimizing the impact of historical embedding with a long time.

## 5. Experiment

In this section, we conduct extensive experiments on benchmark datasets to compare the performance of the DyLFG and baseline methods. Then, the validity of our model is evaluated by analyzing the experimental results.

### 5.1. Datasets

We evaluated the performance of the dynamic network embedding using link prediction as the criterion. Three datasets were used in our experiment, and their statistics are listed in Table 1.

Enron [44] is a public dataset of communication networks based on email records between employees of the Enron corporation. The network consists of 16 snapshots, with an ever-changing number of nodes in each snapshot. Nodes in the network represent the core employees of Enron, and edges represent the occurrence of an email from one person to another. Edge weights in the network represent the number of emails sent between corresponding employees. The number of nodes in this dataset increases over time, but the number of edges sometimes decreases and sometimes increases.

UCI [45,46] is a dynamic network of 13 network snapshots, built on the records of UC Irvine students sending messages on the online platform. We took twelve snapshots out of them to form the dynamic network. Nodes in the network represent student users of the online platform, and edges represent the occurrence of messages sent between peer users. Like Enron, the weight of an edge in the UCI network represents the total number of messages sent between users. The number of nodes in the UCI dataset is increasing in terms of time, while the number of edges is not.

AS733 [25] is a dataset of 733 daily instances of autonomous systems, which is presented in snapshot form. We took sixteen snapshots out of them to form the dynamic network. It should be noted that the number of nodes and the number of edges in the AS733 dataset are not monotonic with respect to time.

### 5.2. Baseline Methods’ Setup

We selected two static-network-embedding methods and two dynamic-network-embedding methods as the baseline methods to compare the performance with our model DyLFG. In order to help the static-network-embedding methods obtain the historical information of the dynamic network, we aggregated the snapshots before time t into snapshot $G_{t}$. The weights of edges in the network obtained by the aggregation are the cumulative sum of the edge weights in all snapshots up to time *t*.

*Deepwalk* [12]: This samples the structure around the target node in the network by truncated random walks and, then, inputs the sequences of nodes obtained by sampling into the skip-gram model. The skip-gram model uses a fixed-size sliding window to obtain the generalized neighbors of the target node and, then, achieves network embedding by maximizing the probability of the co-occurrence of the generalized neighbor pairs.

*Node2vec* [18]: In order to capture the structural equivalence and homogeneity in the network, this performs breadth-first sampling and depth-first sampling by biased second-order random walks. In this way, the node sequences obtained by random walks may contain rich network information, which can improve the performance of network embedding.

*DynWalks* [47]: This designs a node-selection scheme based on the global topology and the recent changes in the network snapshots and, then, learns the embedding dynamically and efficiently according to the selected nodes.

*DySAT* [23]: This extracts the structural information of the network snapshots through the graph attention networks to obtain the corresponding snapshot embeddings and, then, inputs it into the temporal attention layer. The temporal attention layer assigns different importance to the inputs of different time steps and aggregates these inputs to obtain the embeddings containing both structural information and network dynamics.

*DynGEM* [20]: The core of the algorithm is the deep autoencoder, and considering that the number of nodes in different network snapshots is variable, the model dynamically determines the hidden dimension required for each snapshot.

*Dynnode2vec* [17]: To deal with dynamic networks, this algorithm improves Node2vec by taking the previously learned embedding vectors as the initial weights of the skip-gram model and updating the skip-gram with evolutionary random walks.

### 5.3. Link Prediction

Link prediction is used to predict which links between nodes in the real network are more likely to appear or disappear in the near future. Links have a wide range of representative meanings in the real world; for example, they can represent the interaction between users in a communication network, or they can represent the friendship relationship in a social network. The dynamic changes in links reflect the evolution of these real-world networks over time. If the model has good link prediction performance, it shows that it has captured the law of real network evolution.

In this section, we use link prediction as an evaluation metric to compare the performance of the DyLFG and baseline algorithms. Specifically, we used snapshots $\left\{G_{1},G_{2},\cdots ,G_{t}\right\}$ to train the model to obtain node embeddings and, then, used the embeddings as a basis to predict the edges in snapshot $G_{t+1}$. That is, the node pairs in $G_{t+1}$ are classified according to the presence or absence of edges. The AUC [18] and Macro-AUC [23,48] are used as indicators to evaluate the performance of link prediction. The Macro-AUC is the average of the AUC for each time step. In the experiment, the proportion of the training set, verification set, and test set was 20%, 20%, and 60%, respectively. In order to make a fair comparison with the static-network-embedding method in the baseline method, we first aggregated $\left\{G_{1},G_{2},\cdots ,G_{t}\right\}$ into a static network, where the weights of the edges are the sum of the weights of the corresponding edges in the network. For all datasets, the hyperparameter γ in Equation (24) was set to 0.1. For the Enron dataset, the hyperparameters $\lambda_{1}$ and $\lambda_{2}$ in Equation (18) were both set to 1.0. For the email_uci dataset, the hyperparameters $\lambda_{1}$ and $\lambda_{2}$ in Equation (18) were also set to 1. Finally, for the AS733 dataset, the hyperparameters $\lambda_{1}$ and $\lambda_{2}$ in Equation (18) were set to 0.40001 and 1.0, respectively. The experimental results are listed in Table 2.

Table 2 summarizes the Macro-AUC scores for the DyLFG and other baseline methods. The higher the Macro-AUC scores, the better the link prediction performance of the model is. Table 2 reflects the link prediction results of each model on the dynamic network datasets, where the best performance is shown in bold. As can be seen from Table 2, the link prediction performance of the static-network-embedding methods, namely Deepwalk and Node2vec, is lower than that of the dynamic-network-embedding methods. The reason is that the static-network-embedding methods can only obtain the network structural information, but cannot capture the temporal dynamics in the snapshot sequence. Our model DyLFG achieved the best link-prediction performance on all datasets. This showed that our model can make better use of network geometry to help capture network information, which is helpful for the improvement of the performance.

**Figure 4:**
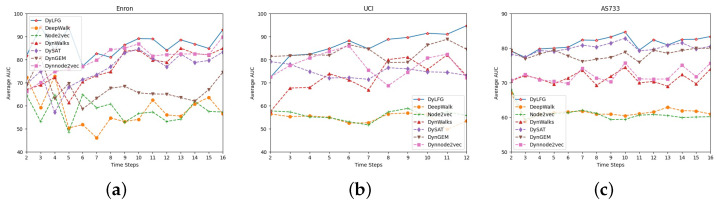
Average AUC of each time step. (**a**–**c**) are, respectively, the average AUC values of each algorithm at each time step for the three datasets.

In addition, as shown in Figure 4, we also compared the AUC value of the DyLFG and other baseline methods at each time step, which reflects the link prediction performance of all methods at each time step, and the higher the value, the better the performance was. As shown in Figure 4a, for the Enron dataset, when the time step is eight, the AUC value corresponding to DyLFG is the second highest, while at other time steps, the DyLFG model corresponded to a higher AUC value than all the baseline methods. As can be seen from Figure 4b, for the UCI dataset, in the listed methods, when the time step was equal to two, the AUC value corresponding to the DyLFG model was the third-highest, that is only lower than the DySAT and DynGEM model. In other time steps, the AUC values corresponding to the DyLFG model were the highest. As can be seen from Figure 4c, for the AS733 dataset, in the listed methods, when the time step was equal to three, the AUC value corresponding to DyLFG was the second-highest, that is only slightly lower than the DySAT algorithm, while in other time steps, the AUC values corresponding to DyLFG were the highest. Compared with Macro-AUC, the AUC value of each time step more specifically reflected the link prediction performance of each algorithm, and the results in Figure 4 show that, on the whole, the link prediction performance of the DyLFG model at each time step was better than the other baseline methods. The model DyLFG easily converged and can be performed in less than 50 epochs of training. However, due to the high computational complexity of the Ricci curvature, the model was not fast compared with the other baseline methods. Whether it is our model or the baseline method with hyperparameters, we used a larger step size for the initial optimization, followed by a smaller step size for the further optimization, to grid search for hyperparameters.

## 6. Conclusions and Future Work

In this paper, we discussed the link prediction problem of dynamic networks based on geometry. Specifically, our DyLFG model uses the GAT module at the lower level to extract the low-order and high-order structural information of the dynamic network at each time step and, then, outputs the hidden layer representation to the newly designed hyperbolic fully connected layer stacked module, which can make full use of the advantages of the hyperbolic geometry for hierarchical data modeling to fit the network data with an obvious hierarchical structure. The dynamic network is different from the static network in that the network structural information it contains is constantly evolving with time. Therefore, it is necessary to capture not only the structural information of the network snapshot corresponding to each time step, but also the temporal dynamic regularity contained in the network snapshot sequence. In order to capture this temporal dynamics, DyLFG model establishes corresponding modules at the lower level of the model and at the top of the model. At the lower level of the model, a new RGRU module was used to evolve the weight matrix of the second GAT layer in the GAT module, thereby connecting the GAT modules of each time step horizontally. Through the horizontal flow of information, the module can initially capture the change trend of the network structure with respect to time. At the top of the DyLFG model, the temporal attention layer was used to further extract the temporal dynamics contained in the network snapshot sequence. The experiments showed that our model outperformed the baseline methods of static and dynamic network embedding on the baseline dataset. As for the future direction, our consideration will mainly focus on two aspects. On the one hand, global information should be added to the model for further improvement. On the other hand, due to the high computational complexity of the Ricci curvature, we will consider designing approximate calculation methods of the Ricci curvature and adopting incremental learning methods of dynamic networks to improve model efficiency.

## Figures and Tables

**Table 1:** Dataset statics.

Dataset	Enron	UCI	AS733
Nodes	143	1795	2102
Edges	2852	13,399	4307
Time steps	16	12	16

**Table 2:** Link prediction results.

Methods	Enron	UCI	AS733
	Macro-AUC	Macro-AUC	Macro-AUC
Deepwalk [12]	58.03	54.62	61.63
Node2vec [18]	57.84	55.94	61.03
DynWalks [47]	76.68	72.51	71.32
DySAT [23]	76.12	74.85	80.09
DynGEM [20]	66.97	83.30	78.30
Dynnode2vec [17]	80.12	77.71	72.13
DyLFG	**86.54**	**86.42**	**81.39**

## Data Availability

All experiments use publicly available datasets, and links to them are provided below: Enron: https://github.com/FeiGSSS/DySAT_pytorch/tree/main/data/Enron, email_uci: https://github.com/FeiGSSS/DySAT_pytorch/tree/main/data/email_uci, AS733: https://github.com/houchengbin/GloDyNE/tree/master/data/AS733.
